# Basic Characteristics of Adults with Periodic Fever, Aphthous Stomatitis, Pharyngitis, and Adenopathy Syndrome in Comparison with the Typical Pediatric Expression of Disease

**DOI:** 10.1155/2015/570418

**Published:** 2015-08-18

**Authors:** Marco Cattalini, Martina Soliani, Donato Rigante, Giuseppe Lopalco, Florenzo Iannone, Mauro Galeazzi, Luca Cantarini

**Affiliations:** ^1^Pediatric Clinic, University of Brescia, Piazzale Spedali Civili 1, 25123 Brescia, Italy; ^2^Institute of Pediatrics, Università Cattolica del Sacro Cuore, Largo Agostino Gemelli 8, 00168 Rome, Italy; ^3^Interdisciplinary Department of Medicine, University of Bari, Piazza Giulio Cesare 11, 70124 Bari, Italy; ^4^Research Center of Systemic Autoinflammatory Diseases and Behcet's Disease Clinic, University of Siena, Viale Bracci 1, 53100 Siena, Italy

## Abstract

Autoinflammatory diseases are caused by inflammasome dysregulation leading to overproduction of proinflammatory cytokines and a pathological delay in the inflammation switching off. The progress of cellular biology has partially clarified pathogenic mechanisms behind monogenic autoinflammatory diseases, whereas little is known about the polygenic ones. Although the genetic susceptibility of periodic fever, aphthous stomatitis, pharyngitis, and adenopathy (PFAPA) syndrome is still obscure, the presence of overlapping symptoms with monogenic periodic fevers, the recurrence in family members, the important role played by dysregulated interleukin- (IL-) 1*β* secretion during flares, the overexpression of inflammasome-associated genes during attacks, and, last but not least, the therapeutic efficacy of IL-1*β* blockade strongly indicate a potential genetic involvement in its pathogenesis, probably linked with environmental factors. PFAPA syndrome has a typical inception in the pediatric age, but a delayed onset during adulthood has been described as well. Treatments required as well as effectiveness of tonsillectomy remain controversial, even if the disease seems to have a self-limited course mostly in children. The purpose of this review is to provide an overview of this complex polygenic/multifactorial autoinflammatory disorder in which the innate immune system undoubtedly plays a basic role.

## 1. Introduction

By definition autoinflammatory diseases (AIDs) are characterized by recurrent episodes of inflammation in the absence of autoreactive T-cells and autoantibodies [1]. From the understanding that the so-called monogenic periodic fevers are the prototype of pure AIDs, our knowledge has now expanded to encompass multifactorial and polygenic diseases among AIDs [2, 3]. PFAPA syndrome, along with other disorders, such as Behçet disease [4, 5], recurrent idiopathic pericarditis [6–8], adult-onset Still's disease, and systemic-onset juvenile idiopathic arthritis [9, 10], belongs to the group of acquired AIDs on a potential multifactorial or polygenic basis. The acronym PFAPA, epitomizing the most characteristic symptoms of the syndrome (periodic fever, aphthous stomatitis, pharyngitis, and cervical adenitis), was coined along with the diagnostic criteria in 1989, 2 years after the first description of this condition made by Marshall in 1987 [11]. This clinical entity is characterized by the regular occurrence of high fever (generally >39°C) associated with at least one of three cardinal clinical signs: aphthous stomatitis, pharyngitis, and cervical adenitis. PFAPA syndrome has been well-described in pediatric patients, since in most cases it occurs in young children, arising before the age of 5, and represents the most frequent cause of periodic fever of unknown origin in childhood, at least of rheumatologic interest. Nonetheless, there is now mounting evidence that children older than 5 years may present with the typical picture of PFAPA syndrome, and recent literature has depicted about 40 cases of onset in adulthood [12–14]. This evidence suggests that the age criterion (i.e., age at onset less than 5 years) should not be considered among PFAPA diagnostic criteria and that rheumatologists should be aware of the clinical characteristics of this syndrome in order to suspect and recognize this disease in their adult patients too.

## 2. Pathogenesis

The exact pathogenesis of the disease has yet to be recognized. Given the dominating symptoms, the occurrence in the first years of life (when upper respiratory tract infections are very frequent), and the efficacy of tonsillectomy, an infectious etiology of the syndrome was firstly proposed [11]. On the other hand, the lack of seasonal clustering and the observation that pharyngeal and tonsil samples were invariably negative for pathogens have led to partially abandoning this theory [12].

More recent theories, evoked by the response to corticosteroids and clinical overlapping with AIDs, have suggested an immunologic dysregulation [15, 16]. The involvement of tonsils made some authors look for specific histologic findings [17]. Petra et al. investigated paired tonsils and peripheral blood samples from 10 children with PFAPA syndrome, who successfully recovered after tonsillectomy. Most of the observed changes in distribution of B and T lymphocytes, together with an elevation in gene expression of T-cell chemoattractants, were restricted to tonsils, suggesting recruitment to this site from the peripheral blood, via impaired chemokine expression [18]. These results were in accordance with another study, which found more IgD-armed basophils (which are thought to play a role in the balance between immunity and inflammation) in the tonsils of PFAPA patients, compared to controls [19]. Even though preliminary, these data altogether point to the tonsils as central in the PFAPA pathogenesis and might partly explain the therapeutic success of tonsillectomy in these patients.

It has been recently observed that several cytokines are elevated during febrile episodes of PFAPA syndrome, primarily interferon-gamma, tumour necrosis factor, interleukin- (IL-) 6, and IL-1*β*. The latter seems to be significantly secreted by dysregulated monocytes during febrile flares and acts as stimulator of T-cell function [20]. The central role of IL-1 in PFAPA patients has been demonstrated also by Stojanov et al., who further underlined the similarities between PFAPA syndrome and hereditary periodic fevers (HPF) [21]. The high incidence of family members with a history relevant to PFAPA syndrome suggested that the disease could be genetically determined, but the exact gene is far from being identified [22–24]. Hypomorphic mutations in genes related to HPF have been described in PFAPA patients. Although this finding needs to be confirmed in larger studies, it may suggest a general model for PFAPA syndrome in which a microbial trigger might give rise to the activation of innate immune system and recruitment of activated T-cells in a susceptible host [25].

In conclusion, even though the exact pathogenic mechanism underlying PFAPA still remains unknown, the clinical overlapping with monogenic HPF, the high rate of positive familial history in patients, the clearly central role of proinflammatory cytokines, mainly IL-1 among them, and the variable association with some hypomorphic mutations of genes involved in HPF all point in our opinion to the idea that PFAPA syndrome should be considered a polygenic autoinflammatory disease [26–28].

## 3. Epidemiology

Although PFAPA syndrome is the most frequent cause of recurrent fevers in children, at least of rheumatologist concern, few pieces of data about its incidence can be found in the medical literature. The largest cohort of patients derives from an international database reporting 301 patients [14]. In a retrospective study carried out by Barbi et al. in Friuli Venezia Giulia (a northern region in Italy) and involving 20 pediatricians, 40 cases of PFAPA syndrome fulfilling the diagnostic criteria were detected during a 5-year period (1996–2001): the approximate incidence was 0.4 cases/1000 children/year, with 1 new diagnosed case of PFAPA per pediatrician every 1-2 years [29]. In a recent paper published by Førsvoll et al. the incidence of PFAPA syndrome in Norway was calculated to be 2.3 per 10.000 children up to 5 years of age [30]. As far as our experience is concerned, we diagnosed 268 children with PFAPA syndrome, observing an ascending climax in the distribution of cases per year: 2/3 of patients were evaluated in the period 1999–2010 and 1/3 during the period 2010–12, testifying that pediatricians are becoming more aware of this syndrome. Data on the PFAPA incidence in adults are completely lacking.

Episodes of fever in PFAPA syndrome generally begin between the ages of two and five years [1, 15, 31]: there is a slight male predominance, but no predilection for particular ethnic groups [14]. Once considered peculiar for children younger than 5, PFAPA syndrome is now known to affect older children and adults as well. Padeh et al. described a group of 15 adults with PFAPA clinical features [12]. More recently, we described 17 adults with similar features of unexplained recurrent fever, satisfying PFAPA criteria [16]. In adults fulfilling the criteria for PFAPA syndrome no gender predominance can be detected (Table 1). Even if no genetic abnormalities have been described, a positive familial history is referred in up to 40% of patients with PFAPA syndrome [14, 22–24].

## 4. Clinical Features

As the PFAPA acronym stands for, fever is the dominant symptom in children and the clinical element that prompts child's parents to ask for medical advice. Body temperature usually increases up to 40.5°C, lasts for 3–5 days, and is quite resistant to the normally used antipyretics, such as acetaminophen or ibuprofen. The most frequent accompanying sign is the presence of erythematous or exudative pharyngitis (i.e., present in up to 90% of patients), cervical lymphadenopathy, with swollen and tender lymph nodes (in up to 75% of patients), and oral aphthosis (in up to 50% of patients) [11, 14, 15]. The clinical picture may be enriched by the presence of headache, abdominal pain, nausea, vomiting, chills, malaise, myalgia, and arthralgia. Some of these “minor” symptoms, such as abdominal pain, may indeed become prominent in the clinical picture of some patients [32]. The episodes recur very regularly with stereotyped clinical characteristics in the majority of patients, giving the impression that there is a “clockwork” mechanism in the episodes. Even though clockwork periodicity is not exclusive for PFAPA syndrome and may not be present in a minority of children, its presence combined with complete wellness between febrile episodes and normal growth and development are nodal points for diagnostic purposes [11]. Nonspecific symptoms such as malaise, irritability, and fatigue may be evident during days before PFAPA flares in up to a third of patients, and parents learn to interpret those clinical signs as prodromes of the PFAPA febrile attack, helping them in differentiating fever from other febrile episodes secondary to infections [15, 32]. The clinical characteristics of adult patients are similar to those described in children, with the exception that arthralgia and myalgia seem to be more frequent in adult patients than in children, while aphthae and chills appear to be less common [12]. Table 1 compares the clinical characteristics of all adult PFAPA patients reported in the literature with PFAPA patients followed up at our centre in the last 15 years. The 3 cardinal signs (aphthosis, pharyngitis, and cervical adenitis) were concurrently present during attacks in 47% of adult patients versus 27% of children; at least 2 cardinal signs were present in 52% of adult patients versus 46% of children; the mean number of episodes/year was 8.3 ± 5.2 in adult patients and 13.3 ± 9.2 in our PFAPA population.

The combination of PFAPA syndrome with autoimmune manifestations has been recently described on an individual basis. Cazzato et al. identified an adult-onset PFAPA syndrome associated with endocapillary proliferative glomerulonephritis, while Corte et al. reported a case of an 18-month-old girl with PFAPA syndrome associated with type 2 autoimmune hepatitis, both responsive to corticosteroid therapy [33, 34]. Recently a case of an 11-year-old boy diagnosed with PFAPA syndrome who developed recurrent acute aseptic encephalitis was described by Frye [35]. Finally Iba et al. described a 7-year-old boy who was diagnosed with PFAPA syndrome at the age of 3, who developed acute parapsoriasis (pityriasis lichenoides and varioliformis acuta, also known as PLEVA) when he was 5 years old [36]. These associations may be just occasional and, to date, we do not have enough clinical or pathogenetic data to establish whether the immune dysregulation in PFAPA patients may predispose them to autoimmunity. Nonetheless, the concurrence of clinical manifestations typical of both AIDs and autoimmune disorders is evocative of the recently developed concept of an immunological continuum, in which diseases lie on a spectrum from autoimmune to autoinflammatory, and the prevalence of an autoimmune versus an autoinflammatory response in the single patient determines the clinical picture [37, 38].

## 5. Laboratory Findings

No diagnostic tests for PFAPA syndrome are available today. During PFAPA flares patients usually display moderate leukocytosis with prominence of neutrophils, while an increase in the number of monocytes, a decrease of eosinophilic rate, and thrombocytosis during the afebrile period have been reported, even though these latter findings need further confirmation and may simply be secondary to the acute-phase response [16]. Erythrocyte sedimentation rate and C-reactive protein are constantly raised during febrile attacks [39, 40]. The recent finding that procalcitonin concentrations do not increase in correlation with the increase of other acute-phase reactants during attacks identifies this protein as a possible useful marker for differentiating PFAPA syndrome from infections [41]. Serum immunoglobulin levels are normal or close to normal, while IgD can be normal or slightly elevated [42]. During episodes all the cultures are negative [43]. In a recent study Yamazaki et al. examined the utility of CD64, a member of the Fc*γ* receptors, in diagnosing patients with PFAPA syndrome. In these patients, CD64 expression on neutrophils and monocytes during the attack-free period was similar to that of controls, while it was dramatically increased on both cell types during flares [44]. Further data confirming this finding are needed before considering CD64 expression as a diagnostic marker for PFAPA syndrome.

## 6. Diagnostic Criteria and Differential Diagnosis

As already pointed out, PFAPA syndrome is a clinical diagnosis. Thomas diagnostic criteria for PFAPA syndrome may be applied to help the clinician in corroborating this diagnosis as follows:

1. Regularly recurring fevers with an early age of onset (<5 years of age).
2. Constitutional symptoms in the absence of upper respiratory infection with at least 1 of the following clinical signs:
  - Aphthous stomatitis.
  - Pharyngitis.
  - Cervical lymphadenitis.
3. Exclusion of cyclic neutropenia.
4. Completely asymptomatic interval between episodes.
5. Normal growth and development.

Noteworthily, although one of the criteria is the age at onset of less than 5 years, it is now well-established that PFAPA children may be older than 5 at disease onset, and PFAPA syndrome may have its onset in adulthood, meaning that the age criterion should not be considered mandatory for diagnosis.

Based on the current criteria, diagnosis of PFAPA syndrome is made on the basis of clinical history, physical findings, and exclusion of other causes of recurrent fevers. For this purpose different diseases have to be excluded, and differential diagnosis may vary on different ages. The first diseases to exclude are infections and, in particular, viral or bacterial pharyngitis. This is usually an easy task to accomplish: the absence of accompanying symptoms related to upper respiratory tract infections is usually enough in the clinical setting to exclude viral infection, while a pharyngeal swab is the gold standard to exclude bacterial pharyngitis. Indeed, to differentiate infections is particularly relevant in younger children, who have the highest incidence of upper respiratory tract infections, but still it is crucial in older patients as well as in adults. The occurrence of typical attacks without a seasonal pattern is another characteristic peculiar to PFAPA syndrome, helpful in ruling out recurrent infections. The second diagnostic step is to exclude cyclic neutropenia (CN), since this is the only clinical entity other than PFAPA characterized by a regular interval between fever attacks [11]. In CN fever usually occurs regularly every 18–24 days and is accompanied by a decrease in the neutrophil count with a nadir of less than 500 cells/mm^3^. Patients with CN may present with pharyngitis and oral aphthosis, but differential diagnosis may be eased by the presence of recurrent respiratory infections and genital ulcers, which are typical of this disease and not of PFAPA syndrome. Finally, fever attacks due to CN do not respond to corticosteroid administration. In case CN is hypothesized serial blood cell count should be obtained once a week for 4–6 weeks, to identify the neutrophil drop and, in case of high suspicion, the genetic test looking for* ELANE* gene mutations is available to confirm the diagnosis [45].

Nowadays, the main challenge for clinicians is differentiating PFAPA syndrome from HPF. The current PFAPA diagnostic criteria demonstrated indeed having very low specificity to this regard. Gattorno et al. showed that a relevant number of children with monogenic periodic fevers also met the diagnostic criteria for PFAPA syndrome and that some of the features considered characteristic of PFAPA syndrome, as clockwork recurrence of febrile episodes, oral aphthosis, and cervical adenitis, may be observed in different HPF as well, particularly at their onset [46]. Moreover, it is now well-established that HPF patients may have their onset in adulthood, and this belated onset is probably secondary to heterozygosity or hypomorphic mutations. In these cases patients may have a milder clinical picture, lacking the most typical clinical characteristics of HPF and further making the differential diagnosis between PFAPA syndrome and HPF difficult [47, 48].

The most frequent disease among HPF is familial Mediterranean fever (FMF), caused by mutations in the* MEFV* gene, encoding a protein named pyrin, which is mainly expressed in the inflammatory cells [1, 49]. Its incidence is particularly high in populations living in the Mediterranean basin (as Armenians, Sephardic Jews, Turks, and Arabs) [50]. Its main clinical features are represented by short and recurrent self-limiting episodes of fever, lasting less than 72 hours, associated with serositis, arthralgia and/or arthritis, and erysipelas-like erythema [51, 52]. In addition, fever attacks are typically prevented by long-term colchicine administration [53]. More than 60% of patients show symptoms during childhood, usually before age of 10, but delayed onset has frequently been reported: 98% of patients show their first symptoms within the third decade. Adult-onset FMF seems to be related to heterozygosity and low-penetrance mutations: because of this genetic condition, adults often experience milder phenotype, whose clinical features tend to be similar to those found in younger patients, except for a lower rate of arthritis and erysipelas-like rash [54]. A diagnostic confirmation might be obtained by* MEFV* genotype analysis.

Mevalonate kinase deficiency syndrome (MKD), also known as hypergammaglobulinemia-D syndrome, is caused by homozygosity or compound heterozygosity in the mevalonate kinase (*MVK*) gene, encoding mevalonate kinase, the first enzyme in the cholesterol biosynthesis pathway [1, 55, 56]. It manifests within the first year of life in 75% of cases and in 100% of cases within the first 5 years: it usually accompanies the whole patient's life, but flares tend to reduce in intensity and become less frequent over time [57]. From a clinical point of view attacks are characterized by high fever lasting 4–7 days and generally recurring every 3-4 weeks, often associated with chills, arthralgia, lymph node enlargement, vomiting, diarrhea, abdominal pain, splenomegaly, and oral aphthae [58]. Cutaneous involvement is very frequent and consists of various types of rashes [59].

Tumor necrosis factor (TNF) receptor-associated periodic syndrome (TRAPS) is related to mutations in the soluble TNF receptor super family 1A (*TNFRSF1A*) gene and is the most common autosomal dominant autoinflammatory disease [60–62]. TRAPS starts with fever attacks lasting even 3 weeks and recurring at variable intervals. In addition to fever, common clinical features that can be detected are periorbital edema, conjunctivitis, migratory erythematous plaques with underlying myalgia, and arthritides or arthralgias [63–65]. Also serosal membrane inflammation can occur, revealing itself through abdominal or thoracic pain and full-blown pericarditis [66]. TRAPS is the most variable entity among AIDs in terms of age of disease onset, frequency, duration, and severity of inflammatory flares, and this heterogeneity is probably linked to the wide spectrum of* TNFRSF1A* mutations. Average patients' age at onset is around 3 years, but adult-onset, frequently related to low-penetrance mutations, has been reported up to the sixth decade. In addition, adult patients with TRAPS may display atypical symptoms, such as idiopathic recurrent acute pericarditis or myocarditis [67–69] and sacroiliitis as unique clinical manifestations [70, 71].

The family of cryopyrin-associated periodic syndrome (CAPS) is linked with mutations in the* NLRP3* gene, encoding a structurally crucial inflammasome protein, named cryopyrin, which directly controls the release of bioactive IL-1*β* [1, 72]. The CAPS family includes three different clinical expressions of mutations involving the same gene, all of them starting in early childhood, with overlapping symptoms: familial cold-associated syndrome (FCAS), Muckle-Wells syndrome (MWS), and chronic infantile neurological cutaneous articular syndrome (CINCA syndrome), respectively, mentioned from the least to the most severe [73, 74]. In particular, FCAS is characterized by fever episodes, skin rashes, and arthralgias, often triggered by exposure to cold temperatures [75], while MWS is characterized by a similar group of symptoms associated with migrating urticaria-like lesions, ocular abnormalities, progressive sensorineural deafness, and risk of amyloidosis [76]. Skin rash in combination with arthropathy, usually involving the knees, and chronic meningeal involvement strikingly characterize CINCA syndrome [77].  Table 2 summarizes the main characteristics of the monogenic AIDs herein described.

How can we distinguish PFAPA syndrome from monogenic AIDs? Given the fact that PFAPA syndrome in children is far more common than HPF, this differential diagnosis is the first step in the approach to a child with periodic fevers of rheumatologic interest, and it is of outstanding priority, since it may avoid unnecessary time- and money-consuming genetic tests. In the majority of cases a careful clinical examination conducted by a pediatrician with expertise in the field of AIDs is sufficient to rule out HPF and confirm PFAPA diagnosis. In this context, the presence of cutaneous eruption, severe gastrointestinal symptoms, arthralgia, and thoracic pain during febrile episodes are the clinical characteristics more typical of HPF compared to PFAPA syndrome, while exudative instead of erythematous pharyngitis is more typical of PFAPA patients [46]. Nonetheless, as above mentioned, younger children with HPF may have a clinical picture strictly overlapping that of PFAPA syndrome. For this reason some authors formulated a diagnostic score, named “Gaslini score,” which may help in identifying those patients at higher risk of carrying a mutation in the genes involved in HPF. Age at onset, positive family history, thoracic and abdominal pain, diarrhea, and oral aphthosis were the variables included in the final model. According to the diagnostic score, a probability of >15% identified patients at high risk of carrying mutations in one of the HPF genes. This tool may be of use in the clinician's hand for differentiating PFAPA syndrome from monogenic hereditary causes of recurring fever [78]. When clinicians tried to apply the pediatric score to the adult population, most subjects carrying mutations can be identified as low-risk patients, thus not in need for any genetic testing. This was probably related to the fact that since the score had been validated for children, advanced age of disease onset played an extremely protective role, together with a phenotype characterized by oligosymptomatic/atypical manifestations due to low-penetrance mutations. Recently, we developed a diagnostic score validated for adult population, with the aim of identifying patients at high risk of carrying mutations in* MEFV* and* TNFRSF1A* genes. MKD and CAPS were not included, because of their early onset in childhood. Early age at disease onset, positive family history for recurrent fever episodes, thoracic pain, abdominal pain, and skin involvement were the variables significantly associated with positive genetic test result and were used to construct the score [79].

In 2011 Pontillo et al. tried to find out a serological marker that could help discriminate monogenic AIDs from PFAPA syndrome: previous studies had described the human glycolytic enzyme *α*-enolase as a potential substrate of caspase-1, whose role in the activation of IL-1*β* through the inflammasome is nowadays well defined. Pontillo's hypothesis was that a high amount of anti-*α*-enolase antibodies (AAE abs) could be found out during processes involving caspase-1 activation. The evaluation of AAE abs showed a difference between PFAPA patients, almost negative for AAE abs, and those with HPF which resulted positive for AAE abs [80]. Unfortunately, to our knowledge, these results have not been replicated in larger case series.

Among polygenic AIDs, PFAPA syndrome and Behçet disease (BD) can overlap, sharing some clinical manifestations, and this could be particularly true in the pediatric age, when Behçet patients may be oligosymptomatic. We have recently hypothesized that patients diagnosed with BD during adulthood could have manifested symptoms compatible with PFAPA syndrome during childhood: therefore, we conducted phone interviews with 80 patients with BD applying Marshall criteria and finding out that a variable percentage of patients fulfilled the diagnosis of PFAPA syndrome in childhood. This could suggest either that a common immune imbalance might underlie both diseases or that PFAPA syndrome may predispose to the development of BD [81, 82]. Of note, these are very preliminary and retrospective data that need to be confirmed on larger numbers; nonetheless they indicate that BD should be ruled out in a patient with PFAPA-like phenotype, particularly in the pediatric age [83].

It is quite intriguing that while PFAPA syndrome is the first disease to be ruled out in children with a periodic fever syndrome, given its frequency, PFAPA diagnosis in adult patients is usually considered only after HPF have been ruled out. This approach is certainly due to the fact that, since few years ago, PFAPA syndrome was thought to be exclusive of the pediatric age. Although it is the authors' opinion that the final diagnosis of adult PFAPA syndrome should be undertaken after the exclusion of HPF, this approach will probably change over time, if more adult PFAPA patients will be reported, clearing out that probably this condition is more common than HPF in adults as well.

## 7. Treatment

Treatment of PFAPA syndrome is still a matter of debate and two general considerations have to be taken. First, since the etiology of the disease is unknown, treatment is essentially symptomatic; second, given that PFAPA is a self-limited disease and no long-term sequelae have been described so far, cost-effectiveness of treatment strategies should be very carefully evaluated. Corticosteroids are successfully used during febrile episodes. One or two doses of prednisone (1-2 mg/kg) or betamethasone (0.1–0.2 mg/kg) can dramatically abrupt fever attacks in a few hours. Other accompanying symptoms, however, can take longer to resolve [11, 14] and, obviously, steroid administration does not prevent further attacks [31, 32, 84]. Corticosteroids are effective in almost every child with PFAPA syndrome and, at this dose and with this frequency of administration, do not have any of the well-known effects of chronic steroid therapy. Physicians should be very reassuring on this aspect, since parents of PFAPA patients are often reluctant to give steroids to their children. Steroids seem to be less effective in adult patients: as shown in Table 1, prednisone at the dose of 50–60 mg/day was administered in 33/36 patients, with a complete response in 28, partial response in 4, and ineffectiveness in 1. Whether this discrepancy is secondary to intrinsic biological difference between children- and adult-onset PFAPA syndrome or it is just a matter of finding the right steroid dose in adults needs further investigation. The only “side effect” related to corticosteroid therapy is the free-interval shortening, which can happen in up to half of children, depending on the study analyzed. Nine out of the 33 adults treated experienced a free-interval shortening after steroid administration [12, 13]. There is no clear evidence whether different steroid preparations have different efficacy or safety profile in PFAPA patients.

Prophylactic treatments have also been experimented both with colchicine and cimetidine, though with variable results. Tasher et al. published their experience on 7 patients with PFAPA syndrome, in whom a reduction in the number of episodes was observed with the introduction of colchicine [84]. Of 5 patients from a multicenter cohort treated with colchicine, 2 had a complete response and 3 patients responded partially [85]. Partial efficacy of cimetidine was documented in a minority of patients [14, 86]. The overall evidence on the efficacy of colchicine or cimetidine is very poor, and their use in clinical practice has been hampered by the need of a continuous administration so that these drugs are not routinely used. Lately, the association between vitamin D and PFAPA syndrome in children has been investigated in two independent studies [86, 87]. In both studies vitamin D levels were lower in PFAPA children compared to controls and, in the study by Stagi et al., vitamin D supplementation in PFAPA patients induced a significant reduction in the number of febrile episodes and a shortening of mean duration of episodes in some patients. Although these data are still to be considered as preliminary, they focus the attention on the well-known effect of vitamin D on immune homeostasis.

The role of tonsillectomy in PFAPA syndrome is controversial. In 1989 an initial study reported that tonsillectomy resolved PFAPA symptoms in 4 children [88]. Afterwards, other studies addressed this issue in larger cohorts of patients [17, 89–93], reporting a wide variability in success rates. Renko et al. randomized 14 PFAPA patients to tonsillectomy and 12 PFAPA patients to observation without surgery. PFAPA syndrome resolved immediately in all 14 patients randomized to tonsillectomy; however, the syndrome resolved spontaneously within 6 months in 6/12 patients who did not receive tonsillectomy [94]. Garavello et al. randomized 39 patients, of whom 19 underwent surgery and 20 were treated with medical therapy. Immediate and complete resolution after surgery was observed in 12/19 cases (63%) [95]. The 18-month follow-up allowed to document the complete resolution of symptoms in all patients who underwent surgery, within 1 year from adenotonsillectomy. In contrast to the findings of Renko et al., spontaneous resolution in the control group was rare, as just 1 patient achieved remission.

A prospective study by Licameli et al. evaluated the long-term efficacy of adenotonsillectomy in 102 patients: 99 experienced complete resolution immediately after surgery. Of the remaining 3 patients, 1 continued having cyclic fevers that were similar in duration and frequency to the presurgery state. The second child, given the lack of response to tonsillectomy, was further investigated and subsequently diagnosed with MKD. The third patient kept having unchanged flares immediately after surgery, but he had complete resolution of the inflammatory episodes within 6 months after tonsillectomy [96].

These differences, in terms of tonsillectomy efficacy, are probably determined by the extreme heterogeneity of the studied populations, in terms of diagnostic criteria adopted, type of intervention (i.e., tonsillectomy versus adenotonsillectomy), and length of follow-up before and after surgery. It is the authors' opinion that tonsillectomy remains a highly efficacious treatment for PFAPA children but since PFAPA syndrome is a self-limiting disease, surgery should be reserved to patients in whom medical treatment with corticosteroids is not a good option (e.g., children with very short free interval between episodes or children with long-lasting disease with parents/child discomfort). Following this approach we sent 62/268 PFAPA to tonsillectomy in the last 15 years: 60/62 patients had complete resolution of the episodes.

Given the fact that PFAPA syndrome in adults seems not to be a self-limited disease, tonsillectomy would be a good option. Unfortunately the available data seem to suggest that tonsillectomy is not effective in adults. Data from the most recent literature (see Table 1) show that tonsillectomy was performed in 16/36 patients diagnosed with PFAPA syndrome in adulthood: the response to surgery was prompt and complete in 1 patient, partial in 2 patients, and ineffective in 13 patients. Of 16 patients who underwent surgery, 4 had tonsillectomy performed during infancy because of recurrent febrile pharyngitis. In 3/4 patients, tonsillectomy achieved a disease-free period of several years before the recurrence of fever episodes, but it did not prevent further development of PFAPA syndrome in adulthood. These observations are very interesting and lead us to speculate that probably tonsillectomy is efficacious in inducing a temporary remission but that the effect is transient and that reappearance of the disease years later can occur as a consequence of the compensatory hypertrophy involving other lymphatic stations [97]. Further long-term studies on children who underwent tonsillectomy are needed to explore this point.

As data extracted from gene expression studies provided evidence of high level of some inflammasome-related cytokines during PFAPA flares, some authors hypothesized that patients can get benefit from IL-1 inhibitors like anakinra. A small series of 5 children with PFAPA syndrome was treated with a single dose of anakinra on the second day of fever, with a dramatic response in both clinical and laboratory parameters [21]. We recently described a case of a 27-year-old man who was resistant to conventional therapy (corticosteroids, colchicine, and tonsillectomy) and was treated with anakinra, with a complete resolution of fever attacks [98]. Although these data are very intriguing, suggesting the central role of IL-1 in PFAPA syndrome, the cost-effectiveness of IL-1 blockers restricts this treatment to very selected cases [99].

## 8. Outcome

PFAPA syndrome is considered a self-limited disease that generally resolves spontaneously before adolescence at least in children [14, 90, 91]. A longer follow-up reported in the literature was made by Wurster et al., who followed a cohort of 59 patients for a period ranging between 12 and 21 years. Spontaneous resolution of PFAPA syndrome was described in 50 patients, while only 9 maintained typical cardinal PFAPA symptoms, though fever was less frequent and faded in severity and duration [90]. Corticosteroids and adenotonsillectomy were effective in modifying the course of the disease. During the follow-up period, no long-term sequelae or other comorbidities did appear. Other studies, with a shorter follow-up, reported a spontaneous resolution in only 20–32% of patients.

No long-term outcome data are available for adult patients diagnosed with PFAPA syndrome: Colotto et al. reported a young woman diagnosed with PFAPA syndrome at the age of 21, whose flares finally resolved after administration of prednisone and for whom no more attacks were observed after a 5-year follow-up [97].

## 9. Conclusive Remarks

PFAPA syndrome is a cause of recurrent fevers of autoinflammatory origin. Once thought to be exclusive of the pediatric age, it is indeed one of the commonest causes of periodic fevers and it is nowadays clear that the disease may have its onset in adulthood as well. Clinical features of PFAPA adults are overlapping with those of PFAPA children. The main difference is that, so far, it is not known whether adults with PFAPA syndrome may spontaneously undergo clinical remission, and tonsillectomy does not seem to be a valid option in these patients. Anyway, the new description of PFAPA syndrome in adults should increase awareness among clinicians, and from now on this diagnosis should always be considered in any adult with a long-lasting history of recurrent and unexplained episodes of fever.

All physicians should familiarize themselves with PFAPA syndrome and pediatric rheumatologists add it in the differential diagnosis of AIDs and HPF, in particular. Early recognition of the clinical characteristics will spare time- and money-consuming investigations in these patients. Further studies are needed to better investigate outcome and therapeutic options in the specific category of adults with PFAPA syndrome.

## Figures and Tables

**Table 1:** Comparison between adult PFAPA and pediatric PFAPA syndrome regarding the main demographic and clinical characteristics and response to treatment.

	Adult PFAPA syndrome	Pediatric PFAPA syndrome
Number of patients	36	268
Gender	18 M/18 F	148 M/120 F
Mean age at diagnosis	30.3 ± 9.3	4.8 ± 2.2
Mean age at disease onset	21.6 ± 10.6	2.4 ± 2
Mean number of febrile episodes per year	8.3 ± 5.2 (16/36 adult patients available)	13.3 ± 9.2
Presence of 3 cardinal signs (aphthous stomatitis, cervical lymphadenitis, and pharyngitis)	17/36	74/268
Combination of 2 among 3 cardinal signs	19/36	124/268
Combination of recurrent fever + only 1 cardinal sign	—	70/268
Response to corticosteroids	Complete response in 28, partial response in 4, and ineffective response in 1	Complete response in 226 and partial response in 2
Response to tonsillectomy	Complete in 1, partial in 2, and ineffective in 13 (tonsillectomies performed during infancy included)	Effective in 60 and ineffective in 2
Response to anakinra	1/36 available: complete response	Not administered
Response to thalidomide	1/36 available: complete response	Not administered

**Table 2:** List of the genetic and clinical features of the main hereditary autoinflammatory disorders.

Disease	*Gene* locus	Protein	Inheritance	Clinical features
FMF	*MEFV* 16p13.3	Pyrin	AR	Fever, serositis, arthralgias or arthritides, erysipelas-like eruption on the legs, responsiveness to colchicine prophylaxis, and amyloidosis in untreated or noncompliant patients

TRAPS	*TNFRSF1A* 12p13	p55 tumor necrosis factor receptor type-1	AD	Fever, severe migrating muscle and joint involvement, conjunctivitis, periorbital edema, arthralgias or arthritis, sacroiliitis, serosal involvement, steroid responsiveness of febrile attacks, and risk of amyloidosis

MKD	*MVK* 12q24	Mevalonate kinase	AR	Fever, widespread polymorphous rash, arthralgias, abdominal pain, diarrhea, lymph node enlargement, and oral aphthosis

CAPS				
FCAS	*NLRP3* 1q44	Cryopyrin	AD	Fever, cold-induced urticaria-like rash, conjunctivitis, arthralgias, and fatigue
MWS				Fever, urticaria-like rash, conjunctivitis, arthralgias, neurosensory deafness, and risk of amyloidosis
CINCAs				Fever, urticaria-like rash, uveitis, papilledema, deforming arthritis involving large joints, neurosensory deafness, aseptic chronic meningopathy and hydrocephalus, and risk of amyloidosis

AD: autosomal dominant, AR: autosomal recessive, CAPS: cryopyrin-associated periodic syndromes, CINCAs: chronic inflammatory neurological cutaneous articular syndrome, FCAS: familial cold autoinflammatory syndrome, FMF: familial Mediterranean fever, MKD: mevalonate kinase deficiency syndrome, MWS: Muckle-Wells syndrome, and TRAPS: tumor necrosis factor receptor-associated periodic syndrome.
